# Supplementary figures and images for: In vitro mechanical vibration down-regulates pro-inflammatory and pro-fibrotic signaling in human vocal fold fibroblasts
Source: PLoS One. 2020 Nov 19;15(11):e0241901. doi: 10.1371/journal.pone.0241901 (PMC7676657; doi:10.1371/journal.pone.0241901)

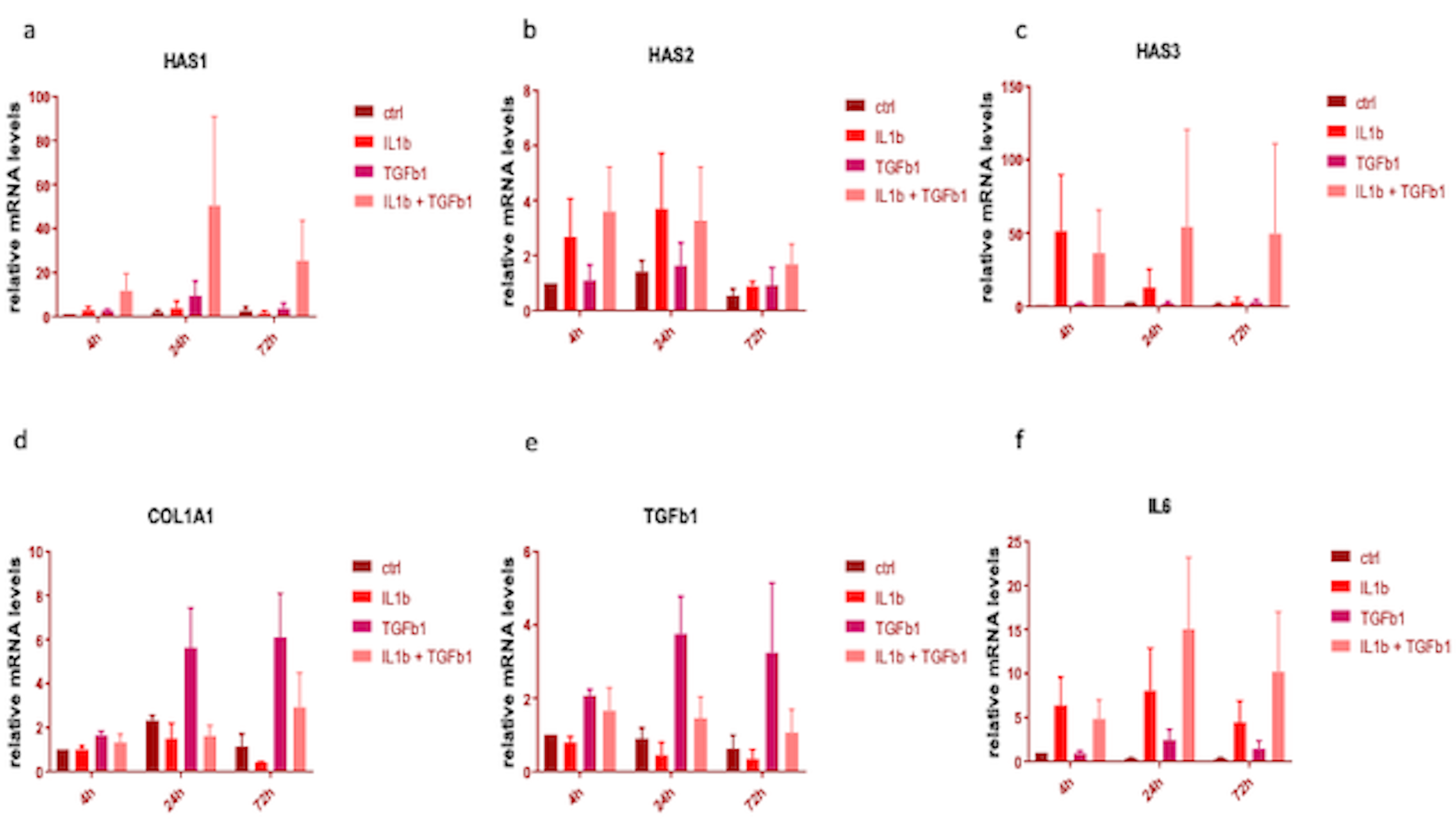

Supplement: S1 Fig — This supplementary figure shows the effect of IL1β, TGFβ1 and their combination on the expression of ECM-related proteins and cytokines over time compared to a non-treated control group. HAS1 (hyaluronan synthase 1), HAS2 (hyaluronan synthase 2), HAS3 (hyaluronan synthase 3), COL1A1 (collagen 1 alpha 1), IL6 (interleukin 6), TGFβ1 (transforming growth factor beta 1). (TIF) [file pone.0241901.s001.tif]
