# Supplementary material for: In vitro mechanical vibration down-regulates pro-inflammatory and pro-fibrotic signaling in human vocal fold fibroblasts
Source: PLoS One. 2020 Nov 19;15(11):e0241901. doi: 10.1371/journal.pone.0241901 (PMC7676657; doi:10.1371/journal.pone.0241901)
Supplement: S1 File — This supplementary file describes in detail the procedure in our previously performed experiments. The presented cytokines in the manuscript were based on these results. (DOCX) [file pone.0241901.s002.docx]

Prior to the present study we conducted trials to evaluate whether TGFβ1, IL1β or a combination of both was the most appropriate to simulate an acute inflammatory reaction. 144.000 hVFF were seeded per well of 6-well plate. After 24 hours the medium was changed to a serum-free medium for starvation. After another day, cells were exposed either to 5ng/mL TGF-β1 or 5ng/mL IL1β or to a combination of both or, for control, to standard medium without any cytokines. Cells were then harvested after four hours, 24 hours and 72 hours. The cell harvesting, RNA isolation and RT-qPCR procedures were identical to the ones of the present study. Gene expression of ECM-related proteins (HAS1, HAS2, HAS3, COL1A1) and pro-inflammatory/pro-fibrotic markers (IL6 and TGFβ1) were measured. The experiment was performed in total three times. Since there were no duplicates in these trials, a statistical analysis would not have been very reasonable and therefore we focused on trends. Even though, the combination of the cytokines did not show the greatest change in each case, in our opinion these results were the most promising.

However, since in these pre-trials other 6-well-plates than in the present study were used and no macromolecular crowding was applied, a direct comparison of the results is not possible.
