# Supplementary material for: In vitro mechanical vibration down-regulates pro-inflammatory and pro-fibrotic signaling in human vocal fold fibroblasts
Source: PLoS One. 2020 Nov 19;15(11):e0241901. doi: 10.1371/journal.pone.0241901 (PMC7676657; doi:10.1371/journal.pone.0241901)
Supplement: S3 File — (PDF) [file pone.0241901.s004.pdf]

3. Material and Methods

3.1. Material

3.1.1. General laboratory equipment

Product	Company	CAS/Ref
Cell culture flask 250 ml 75 cm²	Greiner Bio-One GmbH; Germany	658175
Cell culture flask 50 ml 25 cm²	Greiner Bio-One GmbH; Germany	690175
Bioflex® culture plate + pronectin, C557155	Flexcell® Int. Corp.; USA	BF3001P
Nunclon Multidish 24 well	Thermo Scientific; Denmark	142475
Serological Pipette 5 ml	Corning Incorporated; USA	4051
Serological Pipette 10 ml	Corning Incorporated USA	4101
Serological Pipette 25 ml	Corning Incorporated; USA	4251
ART® 10 µl Barrier Tip, sterile	Thermo Scientific, USA	2140
ART® 20 µl Barrier Tip, sterile	Thermo Scientific, USA	2149P
ART® 100 µl Barrier Tip, sterile	Thermo Scientific, USA	2065E
ART® 200 µl Barrier Tip, sterile	Thermo Scientific, USA	2069
ART® 1000 µl Barrier Tip, XL, sterile	Thermo Scientific, USA	2079-05-HR
Cell Scraper, sterile	Corning Incorporated; USA	3010
Falcon 15 ml Polypropylene Conical Tube	Corning Science; Mexico	352095
Falcon 50 ml Polypropylene Conical Tube	Corning Science; Mexico	352070
Milli-Q® Reference A+ Water Purification System	Merck KGaA, Germany	
Millipak® Express 0.22 µm	Merck KGaA, Germany	MPGP02001
CombiTips advanced 10 ml	Eppendorf, Germany	0030089464
CombiTip 5 ml	Eppendorf, Germany	0030089456
Ethanol denatured ≥99.8 %	Roth GmbH &Co KG; Germany	K928.3
Nitril® 3000 Gloves	Meditrade	1280
Bacillol 30 Tissues	Hartmann	
0.2 µm filter unit	Whatman™, GE Healthcare UK	10462200

QIAzol® Lysis Reagent	QIAGEN Sciences; USA	56008534
RNase AWAY	Molecular BioProducts; USA	#7003
QuantiTect® Reverse Transcription Kit	QIAGEN GmbH; Germany	64-17-5
Ethanol absolute 99,9 % for analysis, Rotipur®	AustrAlco	1.009.832.511
Chloroform Rotipuran® ≥99 %, p.a.	Carl Roth, Germany	3313.1

3.1.4. LDH Assay

Product	Company	CAS/Ref
PierceTM LDH Cytotoxicity Assay Kit	Thermo Scientific; USA	88954
96 well BRANDplates, clear	BRAND, Germany	781602

3.1.5. qPCR

Product	Company	CAS/Ref
GoTaq qPCR Mastermix (2x)	Promega; USA	A600A
Nuclease-Free Water	Promega; USA	P119E
FrameStar® 384 Well Skirted PCR Plate, Roche Style, Plus qPCR Seal	4titude Ltd.; UK	4ti-0382

3.1.6. Western Blot

Product	Company	CAS/Ref
RIPA Buffer (5x)	Cell Biolabs INC.	AKR-191
SuperSignal ™ West Pico PLUS Chemiluminescent Substrate	Thermo Scientific; USA	34578
Precision Plus Protein ™ Standard Dual Color	Biorad	#161-0374
Tris Buffered Saline (TBS, 10x)	Biorad	#1706435
Tris/Glycerin/SDS buffer (TGS, 10x)	Biorad	#161-0732
Restore ™ PLUS Western Blot Stripping Buffer	Thermo Scientific; USA	46430
Tween ® 20 viscous liquid	Sigma-Aldrich	P1379
Laemmli Sample Buffer (4x)	Biorad	#161-0747
