# Supplementary material for: In vitro mechanical vibration down-regulates pro-inflammatory and pro-fibrotic signaling in human vocal fold fibroblasts
Source: PLoS One. 2020 Nov 19;15(11):e0241901. doi: 10.1371/journal.pone.0241901 (PMC7676657; doi:10.1371/journal.pone.0241901)
Supplement: Attachment — Submitted filename: Response to the Reviewers.docx [file pone.0241901.s007.docx]

Dear Editor and Reviewers,

Thank you again for your effort and time spent to review our manuscript and therefore helping us to improve it. We hope we were able to change it for the better by answering all questions. Kindly find below the answers to your inquiries in the order they were asked.

David Hortobagyi

**Reviewer #1:**
**Comment #1**

Abstract: Although IL-1beta is considered a typical inflammatory stimulus, TGF-beta is a pro-fibrotic stimulus. Line 24 will need to revise to reflect better the roles of these two cytokines.

**Answer #1**

*The following modification was made based on comment #1 in the manuscript, abstract, lines 25-26:*

*Inflammatory and pro-fibrotic stimuli were induced by interleukin (IL)1β and transforming growth factor (TGF)β1, respectively.*

**Comment #2**

Introduction: Some writing edits will be needed. For example. Lines 92-99. The transition from introducing Gaston et al and Titze et al' studies and then jumped to Latifi et al.'s bioreactor is abrupt.

**Answer #2**

*Thank you for this comment. The following modification was made based on comment #2 in the manuscript, introduction, lines 102-111:*

*Besides these ‘two-dimensional’ models, there are some three-dimensional approaches as well. Gaston et al. and Titze et al., for example, developed three-dimensional scaffolds where cells are predominantly exposed to tensile stresses.*

*Conversely, Latifi et al. developed a bioreactor, which is currently closest to the in vivo situation. Their intention was to imitate all mechanical forces, VFF are exposed to during phonation. They injected a scaffold, consisting of human VF fibroblasts (hVFF), into silicone VF replicas. Cells were exposed to mechanical forces by applying an airflow from beneath which creates a similar mode of vibration to that of the human VF in the larynx.*

**Comment #3**

Introduction. Lines 114-115. It is also awkward to mention angiogenic factors in the conclusive statement without reviewing the roles of angiogenic factors in the earlier paragraphs.

**Answer #3**

*Thank you for this valuable comment. The following paragraph was included based on comment #3 to the manuscript, introduction, lines 50-52:*

*Since these processes increase the energy metabolism of the tissue, a sufficient blood and consequently nutrient supply is essential. This is enabled by angiogenic factors, which contribute to vessel formation.*

**Comment #4**

Methods: Line 205. Suggest to remove the word "novel" because the work is already published. If the authors would like to retain the word, they need to justify further what the novelty of their bioreactor was in the paper.

**Answer #4**

*The following modification was made based on comment #4 in the manuscript, materials and methods, lines 218-219:*

*Mechanical stimulation was performed using a phonomimetic bioreactor developed and established by our group.*

**Comment #5**

Methods: Line 304. Fix grammatical errors on: "for all other analytes, samples were used undiluted." Also the rationale of not doing validation experiments was weak. As every Luminex plate and experiments may have technical variability, the assumption from previous experiments shall not be applied directly to new experiments. Suggest to run conventional ELISAs for 1-2 markers to confirm the Luminex results.

**Answer #5**

*Thank you for this valuable comment. Just to clarify: Of course, we prepared new standard curves for each protein to enable calculation of the concentrations for each assay, according to the manufacturer’s protocol. Only the sample dilution to reach a concentration within the standard curves was estimated from previous studies.*

*Additionally, we added the standard curves to the supplementary appendix.*

*Following modification was made based on comment #5 in the manuscript, materials and methods, lines 309-316:*

*Standards for each analyte were provided by the manufacturer and included in the kit. These standards were used to prepare standard curves (see supplementary appendix). Additionally, SM without cytokines and SM with cytokines was used as blank, respectively. The sample dilutions for the selected markers were estimated from previous Luminex measurements (e.g. Gugatschka et al 2019, Grossmann et al. 2020). For COL1A1, FN1 and TIMP1 measurement, samples were pre-diluted 1:50; to determine all other analytes, samples were undiluted.*

**Comment #6**

Results: The presentation/ writing will still need to improve. Often times, when reporting certain genes/ proteins have a significant or insignificant change, the authors did not mention the change in relative to which condition/ group. It causes lots of ambiguity in interpreting the results.

**Answer #6**

*The wording of the section results was discussed again and explanations added where we saw any ambiguity (changes are marked in red). The interpretation of the data in combination with the corresponding figures should now be better understandable for the reader.*

**Comment #7**

Discussion. Lines 555-561. The discussion on the roles of IL11 remains brief and superficial. Also, the subsequent sentence of discussing mechanical stimulation and anti-inflammatory effects seemed to be irrelevant. The logical flow of the argument herein needs to be strengthened further.

**Answer #7**

*Thank you for this important comment. We expanded on the roles of IL11. The subsequent sentence was left unmodified since in our opinion it is essential that mechanical stimulation may have anti-inflammatory effects. The following modification was made based on comment #7 in the manuscript, discussion, lines 570-574:*

*IL11 belongs to the IL6-family. It seems to play a pivotal role in the down-streaming pathway of TGF-β1 and therefore represents an essential component of fibrogenesis, such as in cardiovascular fibrosis. Another recent publication indicates that IL-11 is also a key factor in the development of multiple sclerosis. Consequently, it might be a potential therapeutic target to avoid fibrosis.*

**Comment #8**

Discussion. New studies have revealed the important immunological roles of VF fibroblasts, which should be discussed and cited in the paper. For example:
Foote, Al. G., et al. "Tissue specific human fibroblast differential expression based on RNAsequencing analysis." BMC genomics 20.1 (2019): 308.
King, S. N., et al. "Vocal fold fibroblasts immunoregulate activated macrophage phenotype." Cytokine 61.1 (2013): 228-236.
Li‐Jessen, N. et al. "Cellular source and proinflammatory roles of high‐mobility group box 1 in surgically injured rat vocal folds." The Laryngoscope 127.6 (2017): E193-E200.

**Answer #8**

*Thank you for this comment. We have carefully studied the mentioned publications and included and referenced them in our work.*

*The following modifications were made based on comment #8 in the manuscript, introduction and discussion, lines 42-44; lines 112-119 and lines 556-557:*

*Disruption of the vocal fold (VF) mucosa caused surgically, or mechanically by phonotrauma lead to an upregulation of inflammatory cytokines and enzymes as well as damage associated proteins (DAMP) in the VF.*

*A special phenomenon of fibroblasts in general is their diversity. They can accomplish a plethora of functions depending on their localization. The studies mentioned above support the idea that fibroblasts respond to vibration by altering their extracellular matrix (ECM) production. The VFF is predominantly responsible for the composition of the lamina propria. Additionally, in a recent study, VFF were shown to be one of the major sources of high-mobility group box 1, an important DAMP. This cell type is, therefore, essential for a smooth phonation as well as for wound healing.*

*Particularly, the interaction of VFF and macrophages seem to have a huge impact on the inflammatory response.*

**Reviewer #2:**

**Comment #1**

The new title does not address the concern from the previous review that it was too broad and reads like a review. Please revise this to be study specific. e.g. in vitro mechanical vibration down-regulates pro-inflammatory and pro-fibrotic signalling in human vocal fold fibroblasts.

**Answer #1**
Thank you for this valuable comment. The title was changed to “In vitro mechanical vibration down-regulates pro-inflammatory and pro-fibrotic signaling in human vocal fold fibroblasts”, based on comment #1.

**Comment #2**

Nomenclature: In vivo should be italicized on line 393. Human genes and transcripts should be italicized (line 183, 353, 354, 355, 393, 431, 484, 485, 503, 511, 514, 548). Reword line 490 as nomenclature differs between gene and protein - if you list the full name of the gene/protein this would be fine.

**Answer #2**
*All human genes and transcripts were italicized throughout the manuscript based on comment #2 (all changes are marked in red). We could not understand the comment regarding line 490. However, the full names of all genes and proteins are listed at least once in the manuscript.*

**Comment #3**
Amend line 200 to "After a further 73 hours..."

**Answer #3**

*The following modification was made based on comment #3 in the manuscript, materials and methods, line 213:*

*After a further 73 hours, cells were harvested.*

**Reviewer #3:**

**Comment #1**

The organization of the Introduction could be improved. Some very short paragraphs were added about the various models; consequently, the revised Introduction does not have good cohesion and flow. I worry that this section seems unfocused: perhaps organization by subheadings would help.
For example,
Injury/Inflammation
Vocal rest (vs. vocalization)
Bioreactor systems

**Answer #1**
*Thank you for this very valuable comment. The paragraphs were organized by the addition of subheadings suggested from reviewer #3 (changes are marked in red).*

**Comment #2**

The statement that therapy “acts primarily as an adjunctive therapy” is not correct with respect to benign lesions [line 55]. Therapy is a primary treatment for nodules and preferred over surgery, for example.

**Answer #2**

*Thank you for this comment. The following modification was made based on comment #2 in the manuscript, introduction, lines 61-66:*

*Voice therapy can prevent the development of benign vocal fold lesions and, in some cases (e.g. vocal fold nodules), can also function as primary treatment. However, in the majority of lesions, it acts primarily as an adjunctive therapy to a surgical intervention. In these cases, it is still not clear when it is best to begin with voice therapy, though Tang et al. showed that even a presurgical application has positive effects on subjective voice parameters.*

**Comment #3**
What is the meaning of the “two most prevalent ECM components” of the VF LP being collagen and HA [line 158]? If “most prevalent” means “most abundant”, then this statement is incorrect. HA is often touted as being functionally important; however, it represents less than 1% of the LP (per total tissue protein). Refer PMID: 16514800.

**Answer #3**

*Thank you for this important comment. We agree with the reviewer, that there is a certain ambiguity in the wording “prevalent”. Therefore, we made the following modification based on comment #2 in the manuscript, materials and methods, lines 173-174:*

*Two functionally very important ECM components of the VF lamina propria are collagen and HA.*

**Comments #4 and #5**

The Discussion could use attention to organization and flow also. The authors jump back-and-forth between their findings, some clinical studies, animal studies, a bioreactor study – it is challenging to read and grasp the sequence and logic of the authors’ arguments here. It is especially important to take care because the authors shouldn’t overinterpret their data – which reflect in vitro cell phenotypes under certain conditions (admittedly in a sophisticated experimental set-up) – by implying that they are directly comparable to the results of a clinical vocal rest study, for example. Perhaps subheadings would help here also.

The addition of the paragraph on serum starvation is welcome, however it is jarring and seems out-of-place so early in the Discussion. It seems a more natural fit alongside the limitations of the work, later in the Discussion section.

***Answers #4 and #5***

*Thank you for these suggestions. The paragraphs were reordered and the following subheadings were added based on comments #4 and #5 (changes are marked in red): In vivo studies; in vitro studies; Effects of vibration and cytokine-stimuli on VFF.*
