# Supplementary material for: In vitro mechanical vibration down-regulates pro-inflammatory and pro-fibrotic signaling in human vocal fold fibroblasts
Source: PLoS One. 2020 Nov 19;15(11):e0241901. doi: 10.1371/journal.pone.0241901 (PMC7676657; doi:10.1371/journal.pone.0241901)
Supplement: Attachment — Submitted filename: Response to the Reviewers.docx [file pone.0241901.s008.docx]

Dear Editor and Reviewers,

Thank you again for your effort and time spent to review our manuscript and therefore helping us to improve it. Kindly find below the answer to the comment.

David Hortobagyi

**Reviewer #2:**
**Comment #1**

The authors edited protein symbols (transcripts = cDNA/RNA, not proteins) during the last revision and they now read as genes. Correct nomenclature for human genes and proteins is as follows:
Gene/RNA/cDNA - italicize and capitalize gene symbols. You are not required to italicize the gene if you write out the full name.
Protein - capitalize protein symbols (no italics)
The Hugo Gene Nomenclature Committee website has a full set of gene names/symbols and the guidelines to follow if further clarification is needed.

**Answer #1**

*Thank you for this valuable and detailed comment.*

*With all genes and proteins the italicized and non-italicized symbols were used, respectively, as suggested from Reviewer#2 and the international nomenclature guidelines (all changes are marked in red).*
